# *FAST^m^C*: A Suite of Predictive Models for Nonreference-Based Estimations of DNA Methylation

**DOI:** 10.1534/g3.115.025668

**Published:** 2015-12-14

**Authors:** Adam J. Bewick, Brigitte T. Hofmeister, Kevin Lee, Xiaoyu Zhang, David W. Hall, Robert J. Schmitz

**Affiliations:** * Department of Genetics, University of Georgia, Athens, Georgia 30602; † Institute of Bioinformatics, University of Georgia, Athens, Georgia 30602; ‡ Department of Plant Biology, University of Georgia, Athens, Georgia 30602

**Keywords:** epigenetics, DNA methylation, whole-genome bisulfite sequencing, methylome, modeling

## Abstract

We describe a suite of predictive models, coined *FAST^m^C*, for nonreference, cost-effective exploration and comparative analysis of context-specific DNA methylation levels. Accurate estimations of true DNA methylation levels can be obtained from as few as several thousand short-reads generated from whole-genome bisulfite sequencing. These models make high-resolution time course or developmental and large diversity studies practical regardless of species, genome size, and availability of a reference genome.

Advances in high-throughput sequencing has allowed for single-base resolution analysis of DNA methylation at cytosines across an entire genome. This was first applied to the model plant *Arabidopsis thaliana* (Cokus *et al.* 2008; Lister *et al.* 2008) and, since then, has been applied to numerous species, including protists, fungi, insects, anthozoa, tunicates, fish, and mammals (Lister *et al.* 2009; Feng *et al.* 2010; Zemach *et al.* 2010). Currently, DNA methylation is profiled genome-wide by deep, whole-genome bisulfite sequencing (WGBS). The use of a reference genome is essential to inform the methylation status at each cytosine reference position, where a thymine *in lieu* of cytosine indicates an unmethylated cystosine (Urich *et al.* 2015). Thus, absence of a reference genome has prevented rapid, genome-wide analysis of DNA methylation for the majority of known species, and is cost-prohibitive for high-resolution developmental or time-course studies in species with large genomes. To date, several methods exist to accommodate the challenges associated with non-reference-based analysis of DNA methylation, but they lack cytosine context sequence specificity (Kuo *et al.* 1980; Fraga *et al.* 2002; Karimi *et al.* 2006).

Here we present *FAST^m^C*, a suite of predictive models that can be used to estimate genome-wide DNA methylation levels at all cytosine sequence contexts without the use of a reference genome. These models assumed a relationship between DNA methylation levels calculated from alignment of WGBS reads to a reference genome (target; m) and from direct assessment from raw WGBS reads (*i.e.*, no alignment to a reference genome) (estimator; $\hat{F}$) (Supporting Information, Table S1). Methylation levels are calculated as the proportion of methylated cytosines to the total number of possible methylated cytosines. The difference between the two variables exists at unmethylated cytosines; the estimator value includes unmethylated cytosines and true thymines when calculating the DNA methylation level. Estimator DNA methylation levels were compared to target levels to determine a relationship, and the strength of which, to confidently predict/extrapolate genome-wide DNA methylation levels for any sample regardless of the availability of a reference genome.

Using publicly available data, for species with reference genomes, actual and estimator DNA methylation levels for 44 species were used to construct models capable of predicting genome-wide levels of DNA methylation for species without a sequenced genome. Using additional publicly available data from mutants and cell-types known to be different from wild-type samples, we discuss the sensitivity, robustness, and utility of the models in terms of CpG DNA methylation, followed by plant- (CHG and CHH) and mammal-specific (CH) DNA methylation.

## Materials and Methods

WGBS data were downloaded from the Short Read Archive (SRA)/Gene Expression Omnibus (GEO) or sequenced in-house (Table S1). WGBS data were aligned using methods described in (Schultz *et al.* 2015) to generate “allC” files. The allC files were used to determine target DNA methylation levels, and can be downloaded from GEO under accession number GSE72155. Prior to estimation of predictor DNA methylation levels, WGBS data were trimmed of adaptor sequences using Cutadapt v1.9 (Martin 2011), end-trimmed using Trimmomatic (Bolger *et al.* 2014), and quality filtered using FASTX-toolkit (http://hannonlab.cshl.edu/fastx_toolkit/). Reads of at least 30 bp in length with ≥ 20% of nucleotides having a quality score ≥ 75% were retained. Random sampling without replacement was performed with increasing fold-change from 1 to 10^5^ reads using the program fastq-tools (http://homes.cs.washington.edu/~dcjones/fastq-tools/). Custom Perl scripts were used to sum the number of $C^{m}$ and $C^{?}$ sites for each randomly sampled read, and subsequently to estimate the predictor DNA methylation level at CpG, CHG, CHH, and CH sites (Table S1). A characteristic shared among all studies utilizing WGBS data is the inability to distinguish between 5-methylcytosine and 5-hydroxymethylcytosine (5hmC) (Huang *et al.* 2010). Therefore, levels of DNA methylation represent both forms of methylated cytosines, although it should be noted there is no evidence for 5hmC in plant genomes (Erdmann *et al.* 2014).

Predictive modeling is used to find the mathematical relation between a target, (dependent variable) and various estimators (independent variables); subsequent values of an estimator(s) are used to predict the target variable using the established mathematical relationship between them. The goal of the *FAST^m^C* models was to predict reference-based (target) from non-reference-based (estimator) DNA methylation levels. These models assume that in MethylC-Seq data (Urich *et al.* 2015): (i) all cytosines at CpG, CHG, CHH, and CH sites are methylated; (ii) all thymines at TpG, THG, THH, and TH sites are converted unmethylated cytosines or true thymines; and (iii) all nucleotides are randomly distributed in the genome. Our goal is to estimate the proportion of Cs in potential target sites that are in fact methylated, m, which is

$$m=\frac{\sum C^{m}}{\sum \left(C^{m}+C^{u}\right)},$$ (Equation 1)

where $\sum C^{m}$and $\sum C^{u}$ are the total number of methylated and unmethylated target sites in the genome, respectively. The standard method to estimate m is to determine the values of $\sum C^{m}$ and $\sum C^{u}$ by mapping bisulfite sequence reads to a reference genome. Mapping to a reference allows unmethylated Cs, which are converted to Ts during bisulfite sequencing, to be distinguished from true Ts. Our method estimates m using only bisulfite sequence reads. From the bisulfite sequencing data we calculate $\hat{F}$, which is:

$$\hat{F}=\frac{\sum_{s}C^{m}}{\sum_{s}\left(C^{m}+C^{?}\right)},$$ (Equation 2)

where $\sum_{s}C^{m}$ is the total number of methylated target sites in the sample and $\sum_{s}C^{?}$ is the sum of unmethylated target sites, $\sum_{s}C^{u}$, plus sites that are equivalent to unmethylated target sites, $\sum_{s}T$, after bisulfite sequencing in the sample, *e.g.*, all TG dinucleotides in the case of CpG methylation. With our assumptions, if p is GC content, then for CpG methylation, in a sample of n sequenced bases $\sum_{s}C^{m}$ is expected to equal $\left(\frac{1}{4}p^{2}m\right)n$ (*i.e.*, the product of the frequency of CpG sites, the probability of methylation, and the number of bases sequenced), $\sum_{\text{s}}{\text{C}}^{\text{u}}$ is expected to equal $\left(\frac{1}{4}p^{2}\left(1-m\right)\right)n$ (*i.e.*, the product of the frequency of CpG sites, the probability of no methylation, and the number of bases sequenced), and $\sum_{\text{s}}\text{T}$ is expected to equal $\left(\frac{1}{4}p\left(1-p\right)\right)n$ (*i.e.*, the product of the frequency of TG dinucleotides and the number of bases sequenced). Substituting in Equation 2 implies that the expected value of $\hat{F}$ is mp. With our assumptions, $\hat{F}$ is thus an estimate of the product of the methylation frequency of CpG sites and the GC content of the genome. It follows that $\hat{F}$ divided by the estimated genomic GC content, $\hat{p}$, is an estimate of m. For the other three targets of methylation (CH, CHH, and CHG), it can be similarly shown that $\frac{\hat{F}}{\hat{p}}$ is also an estimate of m at those target sites. We estimate GC content from the frequencies of G nucleotides in the sample because these sites are unaffected by bisulfite treatment. The difference between estimates of GC content from WGBS reads are on average within 4.56% ± 3.52% standard deviations of the true GC content. *FAST^m^C* calculates $\frac{\hat{F}}{\hat{p}}$ from a whole-genome bisulfite sample as an estimate of m the fraction of Cs that are methylated.

Violation of the assumptions can cause inaccuracies in estimating $\hat{F}$. We discuss some of these violations in *Results and Discussion*. In addition, we note that when additional genomic short read data (≥ 500,000 bp) are available, GC content and the frequency of the target site in the genome, *e.g.*, the frequency of CpG dinucleotides, can be directly measured. This can then be used to directly calculate the proportion of target sites that are methylated, m, using the frequency of intact target sites, *e.g.*, CpG, that remain in the bisulfite genome data. These are sites that were methylated and thus escaped C to T conversion.

### Data availability

All data used in this study can be found on the SRA/GEO webpages. Accession identifiers can be found in Table S1.

## Results and Discussion

**Figure 1:**
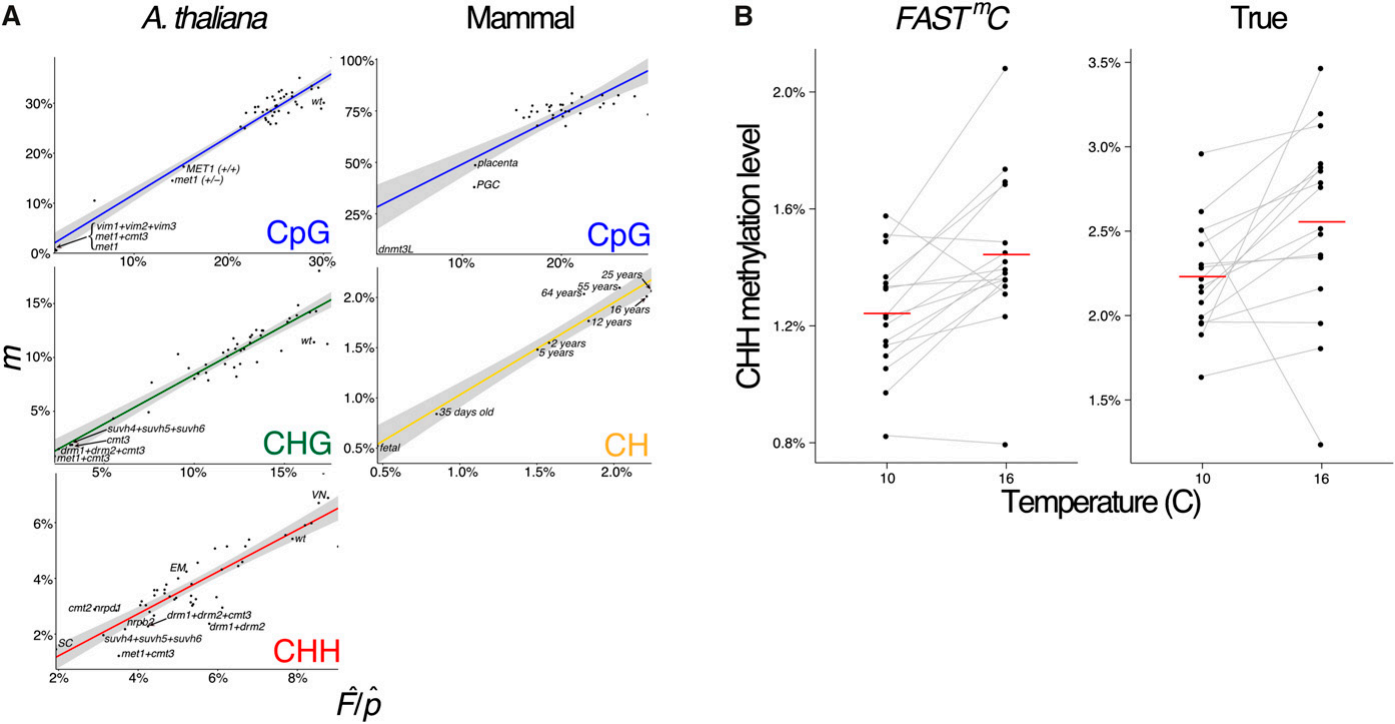
Detection of intraspecific DNA methylation levels by *FAST^m^C*. (A) Linear models (LMs) for estimated levels of methylation, *i.e.*, $\ddot{\hat{F}}/\hat{p}$, (Y-axis) *vs.* actual levels (X-axis) determined by reference mapping of WGBS reads. Estimated levels of methylation were based on 10,000 random WGBS reads. DNA methylation differences between *A. thaliana* mutants, mouse mutants/cell-types/tissues, and increasing CH methylation throughout brain development are captured with *FAST^m^C*. Shaded area represents the 95% confidence interval. (B) Environmental (temperature) effects on CHH DNA methylation in *A. thaliana* is also recapitulated using *FAST^m^C*. Left panel (“*FAST^m^C*”) represents *FAST^m^C* methylation estimates for individual lines based solely on the WGBS reads using the “plant” model from http://fastmc.genetics.uga.edu. Right panel (“True”) represents the methylation values from standard WGBS read alignment to the *A. thaliana* reference genome. Red lines are averages of all lines. Data from Dubin *et al.* 2015.

*FAST^m^C* is able to detect intraspecific differences in DNA methylation (Figure 1). In the plant *A. thaliana*, mutants exist that are defective for enzymes that are required for maintenance of CpG DNA methylation – *met1*, *met1*+*cmt3*, and *vim1*+*vim2*+*vim3* – as they have reduced CpG methylation levels compared to wild-type (Stroud *et al.* 2013). Also, several mutant genotypes for *met1* show different degrees of loss of CpG DNA methylation compared to each other: (i) an original *met1* mutant genotype (high loss); (ii) a *met1* heterozygous mutant genotype (*met1+/−*) (intermediate loss); and (iii) a recovered genotype (*MET1*+/+) from a *MET1*+/+ and *met1*+/− backcross. The recovered *MET1*+/+ is wild-type for MET1 function but has lost CpG methylation in some regions of the genome (low loss). *FAST^m^C* is able to capture the differences between these different genotypes (Figure 1A). Additionally, the slight (∼3%) difference between *MET1+/+* and the *met1*+/− mutant can be distinguished, demonstrating the sensitivity of *FAST^m^C* (Figure 1A).

*FAST^m^C* is also capable of capturing natural epigenetic variation exhibited by changes in levels of CHH methylation due to temperature in *A. thaliana* (Figure 1B). Levels of CHH methylation are affected by temperature such that a higher level is observed at higher temperatures as opposed to lower temperature treatments (Dubin 2015). Applying *FAST^m^C* to these published data (Dubin 2015) using a fraction of the original WGBS data recapitulated these findings (Figure 1B). Thus, studies investigating natural epigenetic variation can be performed at a fraction of the cost.

In mammals, epigenetic reprogramming, including CpG demethylation, is required to erase DNA methylation imprints and epimutations established in the previous generation (Reik *et al.* 2001). Following demethylation, DNA methylation patterns are reestablished at imprinted loci and transposable elements (TEs) during gametogenesis by the *de novo* methyltransferases DNMT3A and a noncatalytic paralog, DNMT3-like (DNMT3L) (reviewed by Law and Jacobsen 2010). The reductions in CpG DNA methylation caused by epigenetic reprogramming in primordial germ cells (PGCs) or by mutations in DNMT3L (*dnmt3L*) compared to somatic tissues are captured by *FAST^m^C* (Figure 1A) (Popp *et al.* 2010; Kobayashi *et al.* 2012; Seisenberger *et al.* 2012). Additionally, increased levels of CpG DNA methylation in the brain (*e.g.*, *NeuN+* and *glia* cells) (Lister *et al.* 2013) can be differentiated from other somatic tissues (Figure 1A and Table S1) (Hon *et al.* 2013). Overall, as demonstrated in *A. thaliana* and *Mus musculus*, *FAST^m^C* can be used to detect intraspecific differences of DNA methylation levels at CpG sites (Figure 1A).

**Figure 2:**
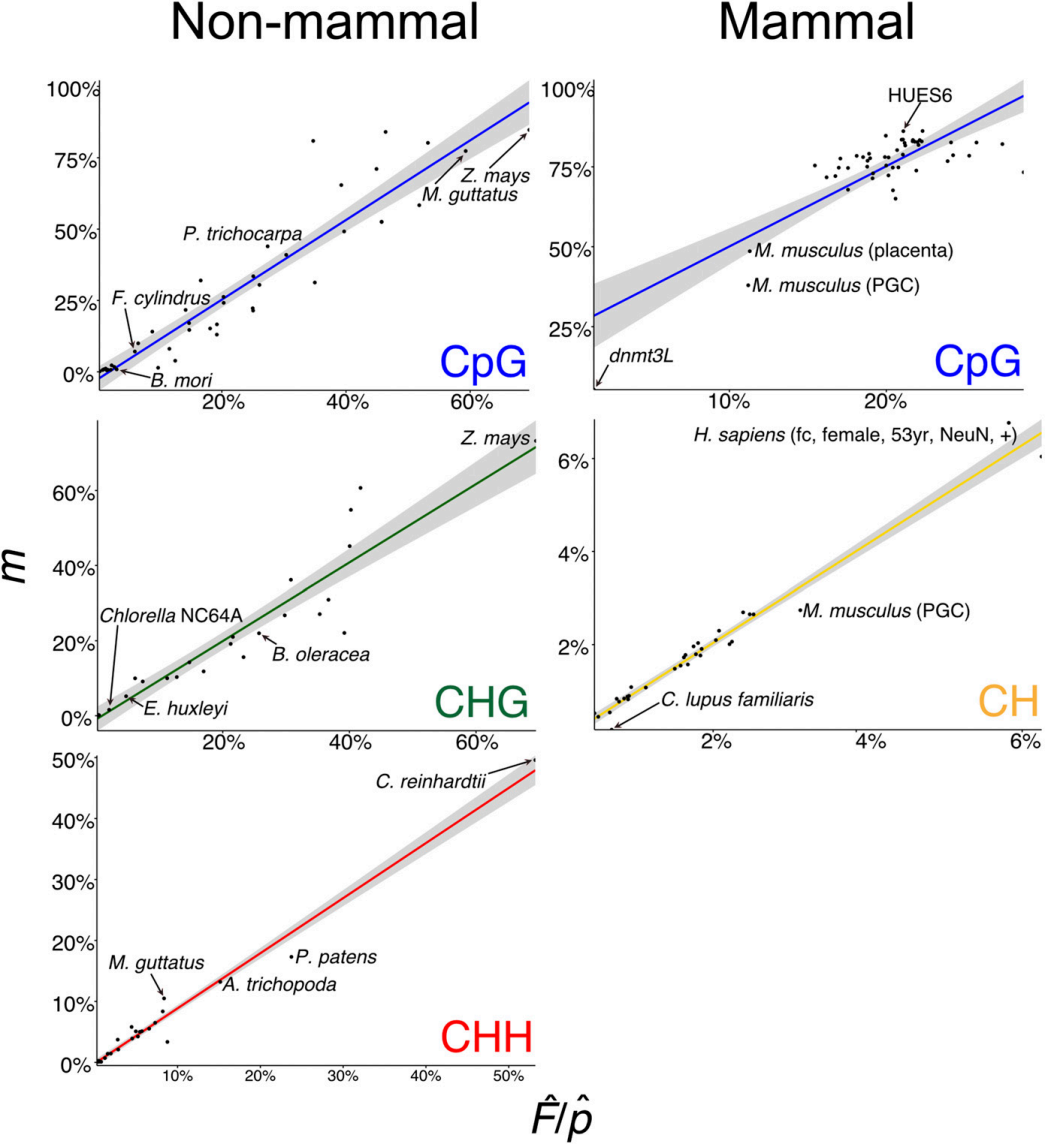
Detection of interspecific DNA methylation levels by *FAST^m^C*. Linear models (LMs) for estimator ($\hat{F}$) *vs.* target (*m*) CpG, CHG, CHH, and CH DNA methylation levels using 10,000 reads corrected for estimated GC content ($\hat{p}$).

We determined natural interspecific variation of DNA methylation at CpG sites across 44 different species (Figure 2). However, unlike intraspecific comparisons between mutants or cell types, nucleotide biases, such as genomic GC content differences, can over- or underestimate the estimator value for the CpG sequence contexts. The estimator (see Equation 2) is estimating the product of the methylation frequency of CpG sites and the GC content of the genome, and is thus confounded. This bias can be overcome in all species investigated but mammals (*Homo sapiens*, *M. musculus*, and *Canis lupus familiaris*) by dividing the estimator value by an average GC content of the genome, which corrects the relationship between target and estimator to ∼1:1. GC content can be approximately estimated from WGBS reads (see *Materials and Methods*) or additional genomic sequence data – 10,000 50-bp reads (500,000 bp) – can be used to directly estimate GC content (Table S1).

Nucleotide biases in genomes – such as the depletion of CpG dinucleotides to localized “CpG islands” in mammalian genomes – may interfere when estimating $\hat{F}$. CpG dinucleotides can be directly measured from 10,000 50-bp genomic sequencing reads (Table S1), and this can then be used to directly calculate the proportion of target sites that are methylated, m, using the frequency of intact target sites, *e.g.*, CpG, that remain in the bisulfite sequencing data. These are sites that were methylated and thus escaped C to T conversion. Accommodating for nucleotide biases in mammalian genomes does not improve assessment of DNA methylation levels by *FAST^m^C* (Table S1). However, treating mammals separately from other species with CpG DNA methylation (*i.e.*, phylogenetic correction) produces an improved, mammal-specific model with similar accuracy – measured as the Mean Absolute Percentage Error (MAPE) – to the remaining species (Table S1). Additionally, only a modest increase in model improvement was observed for nonmammalian species (Table S1). Overall, GC content correction ($\hat{F}/\hat{p}$) and treating mammalian species separately improves model accuracy without introducing additional genomic sequencing data.

*FAST^m^C* also tolerates high contamination and error rates associated with sodium bisulfite conversion. We used WGBS data from *A. thaliana met1* mutants (Stroud *et al.* 2013), which show minor (∼3%) to large (∼14%) differences in CpG DNA methylation compared to wild-type *A. thaliana*. By artificially introducing unmethylated chloroplast reads to 10,000 reads to *met1* and *met1+/−* mutant genotypes, and *MET1+/+* and *A. thaliana* wild-type genotypes, we were able to demonstrate that an ∼3% difference in DNA methylation can still be detected with < 10% chloroplast contamination, and a difference of 13%–14% with 40%–50% chloroplast contamination (Table S1). Similarly, nonconversion rates > 3% still allow for detection of differences between samples (Table S1). It should be noted that the *met1* mutants and *A. thaliana* samples had nonconversion rates of 0.50%, 0.82%, 1.86%, and 0.56% for *met1*, *met1+/−*, *MET1+/+*, and wild-type *A. thaliana*, respectively. The artificially introduced error rates are extremely high, but possible. For example, < 1% of reads typically map to the chloroplast genome, and nonconversion rates are typically < 2% (data not shown). However, it is recommended that Lambda DNA be sequenced for each batch of WGBS libraries prepared to estimate the rate of sodium bisulfite nonconversion. Reducing technical error is especially important for identifying differences between species with small amounts of or no DNA methylation like insects (Table S1). Regardless, the *FAST^m^C* method is robust as it is able to tolerate technical and biological contamination.

The number of short-reads (≥ 30 bp) required to make accurate estimations is low, and we have determined that a few thousand reads produce high-confidence estimates of genome-wide methylation levels (Figure S1). Additionally, very little variation in predicted DNA methylation level is observed [standard error (se) = 0.0013] between 20 replicates of 10,000 randomly sampled reads (Table S1). Thus, these models can be used to accurately and cost-effectively identify differences of DNA methylation levels for any species regardless of the availability of a reference genome assembly.

Non-CpG DNA methylation can also be confidently predicted within and between species using *FAST^m^C*. In *A. thaliana*, the majority of DNA methylation at CHG sites is maintained by chromomethylase CMT3 through a reinforcing loop with H3K9me2 methylation catalyzed by the KRYPTONITE (KYP)/SUVH4 protein (Jackson *et al.* 2002; Du *et al.* 2012, 2014). Similarly to MET1, mutations in CMT3 cause reductions in CHG DNA methylation (Stroud *et al.* 2013), which are accurately detected by *FAST^m^C* (Figure 1A). Also, in *A. thaliana*, cell-type specific levels of CHH DNA methylation in the sperm cell (SC) (*i.e.*, hypo-CHH DNA methylation) and vegetative nucleus (VN) (*i.e.*, hyper-CHH DNA methylation), and depletion of CHH DNA methylation in mutants in the *de novo* DNA methylation pathway (*e.g.*, the DNA-dependent RNA polymerase, POLIV) were recapitulated (Figure 1A) (Calarco *et al.* 2013; Stroud *et al.* 2013).

In mammals, non-CpG DNA methylation can be found at CH sites. A previous study demonstrated an overall increase of CH DNA methylation during brain development in *M. musculus* and *H. sapiens* (Lister *et al.* 2013). *FAST^m^C* was able to capture the overall trend of increasing CH methylation through brain development in *H. sapiens* (Figure 1A). Furthermore, despite only small differences in brain CH methylation in the intervals from 2 to 5 yr (0.068%), and from 55 to 64 yr (0.062%) of age, the *FAST^m^C* model accurately detected these changes (Figure 1A) (Lister *et al.* 2013).

In conclusion, we propose several models that capture the variation of, and can accurately predict, genome-wide DNA methylation levels between species to represent *FAST^m^C*, and these can be found at http://fastmc.genetics.uga.edu. Additionally, the web-based interface makes *FAST^m^C* universally accessible, and models will be continuously updated when new whole genome and methylome data are analyzed and become available. Although genome content biases interfere with the accuracy of *FAST^m^C*, treating mammalian species separately for CpG DNA methylation overcame this obstacle. *FAST^m^C* makes previously intractable studies practical (*e.g.*, high-resolution time course, developmental, and large diversity panels) regardless of species, genome size, and availability of a reference genome. Furthermore, these models will greatly contribute to high-resolution screening of either developmentally or environmentally induced epigenomic reprogramming events. *FAST^m^C* is a suite of powerful models that can aid researchers to make better investments in more comprehensive, fruitful studies.

## Supplementary Material

Supporting Information
