# Supplementary material for: FASTmC: A Suite of Predictive Models for Nonreference-Based Estimations of DNA Methylation
Source: G3 (Bethesda). 2015 Dec 14;6(2):447–52. doi: 10.1534/g3.115.025668 (PMC4751562; doi:10.1534/g3.115.025668)
Supplement: Supporting Information [file supp_6_2_447__index.html]

FASTmC: A Suite of Predictive Models for Non-Reference-Based Estimations of DNA Methylation — FASTmC: A Suite of Predictive Models for Nonreference-Based Estimations of DNA Methylation — Supporting Information 

# *FASTmC*: A Suite of Predictive Models for Nonreference-Based Estimations of DNA Methylation

## Supporting Information for Bewick *et al.*, 2016

**Files in this Data Supplement:**

- Figure S1 - Comparison of estimator DNA methylation levels for (A) CpG, (B) CHG, and (C) CHH between adjacent subsamples. (.pdf, 229 KB)
- Table S1 - This table contains GEO/SRA accession ids for all data used in this study. (.xlsx, 97 KB)
