# Supplementary material for: FASTmC: A Suite of Predictive Models for Nonreference-Based Estimations of DNA Methylation
Source: G3 (Bethesda). 2015 Dec 14;6(2):447–52. doi: 10.1534/g3.115.025668 (PMC4751562; doi:10.1534/g3.115.025668)

Figure S1. Comparison of estimator DNA methylation levels for (A) CpG, (B) CHG, and (C) CHH between adjacent subsamples. As expected, most variation (measured as 95% confidence intervals) in estimates of estimator DNA methylation levels are at low subsamples. However, variation quickly drops, and no significant difference in estimates are observed for ≥10,000 reads.

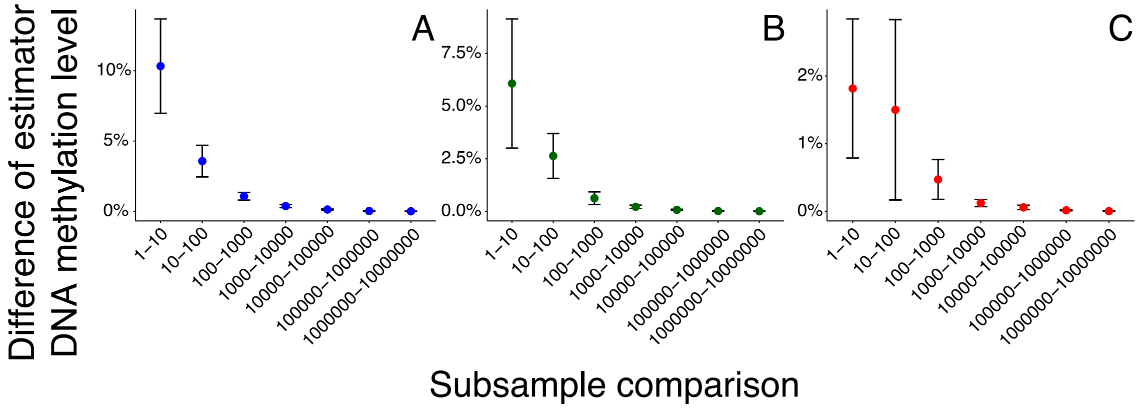

Supplement: Supporting Information [file supp_g3.115.025668_FigureS1.pdf]
